# The Hypodermic Use of the Tincture Veratrum Viride in Puerperal Eclampsia

**Published:** 1877-01

**Authors:** J. W. Griggs

**Affiliations:** West Point, Ga.


### THE HYPODERMIC USE OF THE TINCTURE VERATRUM VIRIDE IN PUERPERAL ECLAMPSIA.

By J. W. Griggs, M.D., West Point, Ga.

It is with great diffidence that I present this communication for publication. Nothing but a sense of duty that I owe to the profession actuates me. Hoping that the importance of the subject will command attention, I more confidently assume the task. In the treatment of a case of puerperal eclampsia, I extemporaneously administered the tincture of veratrum viride by hypodermy, succeeding ultimately in checking the spasms. I have been led to try the virtue of the remedy in several cases since; meeting with happy results, I feel warranted in claiming for it a new interest to the profession. Thus my reasons for writing this paper, setting forth my views, and the better to impress the profession with the effects that I claim, I will append my cases, three in number.

Case I. In March, 1875, I was called in consultation with Dr. Cooper, several miles distant, where he had been tied up with a case of obstetrics from early morning until eight o'clock at night. I sent my instruments in advance, and on my arrival at the home of the patient, was informed by Dr. Cooper that the girl (who was a negress), a primipara, had suffered with convulsions all day. On obtaining instruments, he delivered her of a dead child, but convulsions continued without abatement. He asked me to go in and do what ever I thought necessary for her relief. I found she was having the convulsions at from ten to twenty minutes intervals; pulse from 110 to 115 beats per minute. I administered morphia sulph., gr. $\frac{1}{6}$, and watched the effect, but convulsions continued. I then inserted two drops of veratrum with $\frac{1}{8}$ grain of morphia, by needle syringe. This produced a marked effect. Shortly, however, symptoms of another convulsion appeared. I find no better term to express it than as an abortive spasm. She rested quietly for two hours, when I injected three drops of veratrum, and was gratified at its sedative action. By this time the circulation showed 60 beats per minute. I continued this treatment until three o'clock A.M., when I retired. At seven o'clock patient was perfectly rational. The treatment consisted in keeping the circulation a little below the normal pulse. The morbid symptoms were attributed to albumenuria.

Case II. In September, 1876, my father, Dr. A. W. Griggs, called me to assist him in a case of eclampsia in a patient whom he had delivered six hours previously of a male child at the full term. The patient was a white woman about twenty-six years of age, and had given birth to a child, and suffered an abortion before. When I arrived she was having convulsions every hour regularly, and was entirely unconscious. She had complained greatly of her head; had been well bled, and had taken chloral hydrate, bromide potassium, and cold affusion was applied to the head. There was no abatement of the symptoms from the treatment. The pulse was 100 to 120. We then inserted, by hypodermy, morphia sulph. gr. $\frac{1}{8}$, tinct. veratrum viride gtt. 3. The pulse sank in thirty-five minutes to 90, and she did not have a convulsion in two hours and a half. Then we noticed an increase in the pulse before the attack came on, which was not so severe as former paroxysms had been. After this the circulation was closely watched, and the pulse was kept below 70 beats per minute by the occasional ntroduction of two or three drops of the veratrum hypodermcally. There was no further trouble in the case. The patient gradually recovered consciousness, and no unpleasant symptoms followed. The urine showed albumen in abundance by the usual test.

Case III. Mrs. G., aged 23, a primipara, was taken in labor in December, 1876. My father reached her and was with me about night. The labor progressed as usual, only that the patient complained much of her head at the time of each labor throe, until the head of the child began to descend into the inferior strait, when convulsions came on. The patient was bled freely after the second, but had a very severe spasm while she was bleeding, although she had already lost about sixteen ounces. The attacks came irregularly afterwards, occasionally alternating with labor pains. After morphia gr. $\frac{1}{8}$, tinct. veratrum gtt. 4 was inserted the fourth time, the pulse was reduced below the normal standard, and mustard friction freely used brought partial reaction; consciousness returned after an hour. She still complained of her head. About five hours elapsed, and as the head of the fœtus was pressing upon the perineum, the unwelcome guest returned, and immediately after the second, my father applied the forceps and delivered her of a living male child, and the convulsions ceased. She is making a good recovery.

Before veratrum was used the attacks were every thirty or forty minutes, after its use, the *interval was prolonged to five hours.* Allow me to state that cold water was frequently and freely applied to the head, as suggested by my friend Dr. J. E. McMillan, who, with Dr. J. M. Hatchett, my father's old friend, and a veteran in the cause, rendered invaluable assistance in the case. There was but little albumenuria in this case. The symptoms were most likely those of distributed labor, or of reflected irritation. This case shows that some caution must be observed in the use of veratrum. The amount used should be proportionate to the strength and frequency of the pulse. My father has long been in the habit of using veratrum in puerperal and hysterical convulsions, but I think I am the first to use it in the way described in this paper.

